# The Role of Vitamin D in Reducing the Risk of Metabolic Disturbances That Cause Cardiovascular Diseases

**DOI:** 10.3390/jcdd10050209

**Published:** 2023-05-11

**Authors:** Ziad H. Al-Oanzi, Fawaz O. Alenazy, Hassan H. Alhassan, Yasir Alruwaili, Abdulaziz I. Alessa, Nouf B. Alfarm, Maha O. Alanazi, Sarah I. Alghofaili

**Affiliations:** 1 Department of Clinical Laboratories Sciences, College of Applied Medical Sciences, Jouf University, Sakaka 72388, Saudi Arabia; 2 Department of Pharmacy, Prince Sultan Cardiac Center, Riyadh 11159, Saudi Arabia

**Keywords:** Vitamin D, metabolic syndromes, obesity, hypertension, diabetes and cardiovascular disease

## Abstract

Among the most common problems facing public health today is a lack of vitamin D, which plays a role in the physiological processes of chronic illness conditions. Vitamin D deficiency in metabolic disorders has primary effects on osteoporosis, obesity, hypertension, diabetes, and cardiovascular disease (CVD). Vitamin D acts as a “co-hormone” in the various tissues of the body, and it has been found that vitamin D receptors (VDR) are present on all cell types, suggesting that vitamin D has a wide range of effects on most cells. Recently, there has been a surge in interest in assessing its roles. Vitamin D insufficiency increases the risk of diabetes because it lowers insulin sensitivity, and also raises the risk of obesity and CVD because of its effect on the body’s lipid profile, particularly in terms of the prevalence of dangerously high levels of low-density lipoproteins (LDL). Furthermore, vitamin D insufficiency is often related to CVD and connected risk factors, highlighting the need to know vitamin D’s functions in relation to metabolic syndrome and related processes. Through looking at previous studies, this paper explains why vitamin D is important, how deficiency is related to risk factors for metabolic syndrome through different mechanisms, and how deficiency affects CVD.

## 1. Introduction

Vitamin D, also known as a steroidal co-hormone, is generated by the skin, and it is considered a fat-soluble vitamin. Initially, it was thought to be beneficial for mineral homeostasis and bone health. It controls the metabolism of phosphate and calcium, so it was used to prevent or treat rickets and osteoporosis in children and adults [1]. Recent research has shown that the protective impact of vitamin D is achieved through the binding of vitamin D receptors (VDR) that control gene expression [2,3,4]. In addition, in more recent times, significant consideration has been given to the action of these receptors, as it was found that they are not only in bone cells, but also in other types of tissues, for instance skeletal muscles, vascular endothelial cells, and others, which demonstrates the possibilities of the influence that a lack of vitamin D has on the development of various illnesses, the most notable of which being cardiovascular disease (CVD) [5,6,7,8]. To begin, we need to understand how vitamin D deficiency develops, or what the underlying factors that contribute to its absence are. There are three main ways that lead to deficiency, and they are as follows: sources of vitamin D intake; metabolic processes of vitamin D in terms of absorption, transport, and conversion to the active form; and low vitamin D levels as the result of metabolic abnormalities induced by chronic diseases, including hypertension, diabetes, and obesity. Vitamin D deficiency is assumed to be one of the early stages that may develop in metabolic diseases. Since it has been found that a considerable section of the population is deficient in vitamin D, while at the same time there is a prevalence of obesity globally, there are still doubts about how vitamin D works in terms of preventing CVD [9]. In addition, prior research has connected vitamin D insufficiency to CVD; however, the mechanism by which vitamin D defends against cardiovascular problems has not been completely established [6,7,10]. This review will examine some of the most prominent theorized mechanisms reflecting the association between vitamin D and metabolic syndromes in terms of their impact on CVD. Among the most prominent mechanisms of metabolic disturbances are increased blood pressure through the renin–angiotensin system, lack of improvement in vascular compliance, elevated levels of lipids and especially elevated low-density lipoprotein (LDL) in the blood in obese subjects, and elevated blood glucose in patients with diabetes, as well as elevated inflammatory markers resulting from oxidative stress.

## 2. Vitamin D Metabolism and Action Mechanism

**Figure 1:**
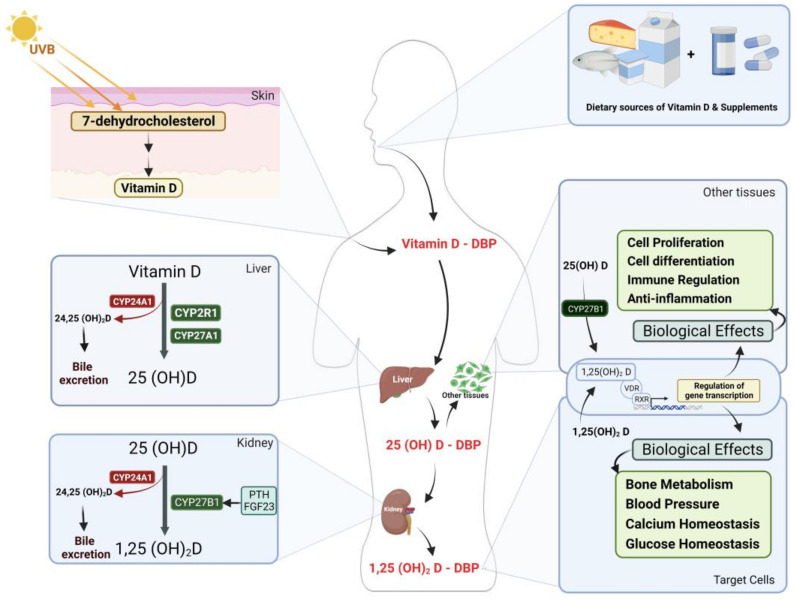
Vitamin D: sources and metabolic processes, and activation mechanisms. Created with BioRender.com.

Under the influence of sunshine and UV radiation, a non-enzymatic process occurs in which one of the fragments of the compound 7-dihydrocholesterol, which is found beneath the skin and is a derivative of cholesterol, breaks off and is converted into vitamin D [11]. The Sun is one of the most significant sources of vitamin D, accounting for up to 80%, while vitamin D-rich foods account for the remaining 20% [12]. Vitamin D is recognized as a fat-soluble vitamin that cannot pass through blood vessels unless attached to a carrier protein. There are two ways to transport vitamin D through blood vessels. The first is via attaching to the specialized protein known as vitamin D-binding protein (DBP), which is highly specialized and has a binding affinity between 85 and 90%. Secondly, there are albumin and lipoproteins, which have a binding affinity of 10–15% or less [13,14]. Vitamin D is carried to the liver through DBP, where the first oxidation of vitamin D occurs, and it is converted into 25-hydroxyvitamin D (25(OH)D) by a group of cytochrome P450 oxidase enzymes (CYPs). Among the most crucial enzymes in vitamin D metabolism to form 25(OH)D is 25-hydroxylase (CYP2R1) (CYP27A1) [15]. In contrast, an enzyme, 24-hydroxylase (CYP24A1), regulates and balances vitamin D levels in the blood by converting it to the inhibitory form 24(OH)D, which is then eliminated through the bile duct and gut. Subsequently, oxidized vitamin D is transformed into 25(OH)D, the most prevalent and abundant metabolite form in the blood that lacks biological action [16]. Through DBP, it is delivered to the kidneys, where the enzyme alpha-hydroxylase (CYP27B1) oxidizes 25(OH)D to form 1,25-dihydroxyvitamin D (1,25-(OH)2D), which is the biologically active form of vitamin D. Additionally, the levels of the biologically active form of vitamin D (1,25-(OH)2D) are controlled and balanced in terms of activating its conversion to the active form by parathyroid hormone (PTH) and 1,25-(OH)2D itself in cases of deficiency, and it is inhibited by fibroblast growth factor 23 (FGF23), as well as the minerals calcium and phosphate [17]. In contrast, conversion to the active form of vitamin D is hindered by the enzyme 24-hydroxylase (CYP24A1) converting it to the excretory form, 24,25-(OH)2D, or other excretory forms, with the ultimate product, calcitroic acid, transported through the biliary system into the gut [16]. The enzyme’s activation by a high concentration of 1,25-(OH)2D, FGF23, calcium, and phosphorus and a low concentration of the enzyme is regulated by PTH. Nevertheless, not only do the kidneys convert the active form of vitamin D to 1,25-(OH)2D, but the great majority of cells in the body also contain the enzyme 1,25-hydroxylase (CYP27B1) and convert it to 1,25-(OH)2D; however, their regulation is distinct from that of the kidneys [16,17,18]. The recent identification of VDR in most body cells has increased interest in the function of vitamin D in the critical activities of different cells [19]. Vitamin D exerts its biological effect when it attaches to the VDR and translocate to the nucleus. Vitamin D promotes gene expression by binding to the retinoid receptor (RXR) and then the gene region known as the vitamin D response element (VDRE) [20]. This stimulates the transcription of vast numbers of genes, which is subsequently followed by the translation process to produce proteins that function in a multitude of biological activities through different mechanisms [21,22] (Figure 1).

## 3. Vitamin D and Cardiovascular Disease Risk Factors: Potential Mechanisms

**Figure 2:**
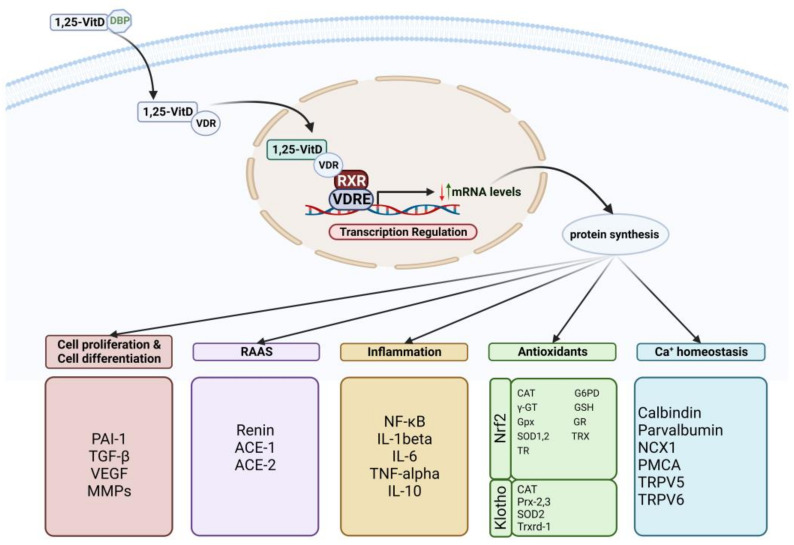
The role of vitamin D and vitamin D receptor (VDR) in the regulation of a vast number of genes located in several cell types to express proteins that act in a variety of cellular functions. Created with BioRender.com.

Vitamin D acts as a co-hormone similar to steroid hormones by binding to the VDR, which is present on a variety of cells in the body, such as heart muscle, vascular smooth muscle, and endothelium cells [6,7,8]. Current data demonstrate that patients with a vitamin D deficiency are at a higher risk of developing CVD [23]. Several processes have been proposed as the means by which vitamin D binds to receptors that are present in cardiovascular cells and activates gene expression to synthesize proteins that work to regulate defects that occurred as a result of metabolic syndromes: high blood pressure through the negative control of RAAS [24,25,26], elevated markers of inflammatory factors caused by oxidative stress [27], lipid levels [28], especially low-density lipoproteins (LDL) [29], calcium levels and its balance in the blood [30], and finally, insulin resistance controlling blood glucose [31]. All these mechanisms are linked together in a single formation that eventually causes CVD (Figure 2).

**Figure 3:**
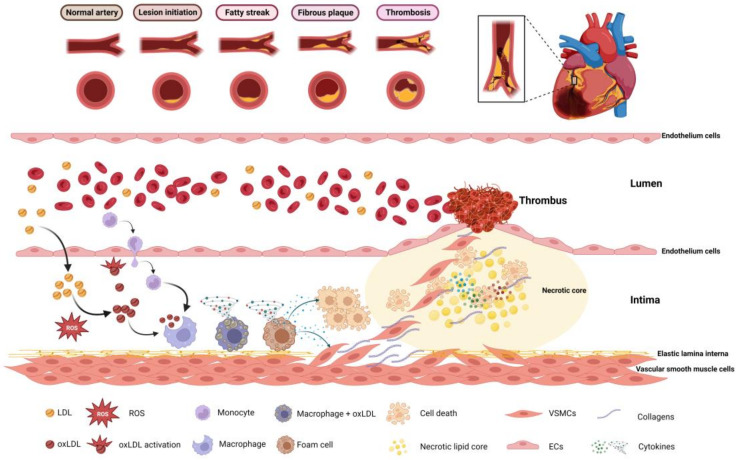
Explanation of the mechanism that contributes to the formation of atherosclerotic plaques. Abbreviations: LDL: Low-density lipoprotein; oxLDL: Oxidized LDL; ROS: Reactive oxygen species; VSMCs: Vascular smooth muscle cells; ECs: Endothelial cells. Created with BioRender.com.

Among the most essential of these vitamin D expressions are Klotho and Nrf2, which perform many of its homologous functions [32,33]. Nrf2 activates gene modifications of a number of antioxidants in addition to enzymes that carry out detoxification; for example, catalase (CAT), gamma-glutamyltransferase (γ-GT), glucose 6-phosphate dehydrogenase (G6PD), glutathione peroxidases (Gpx), glutathione (GSH), thioredoxin reductase (TR), glutathione reductase (GR), superoxide dismutase 1,2 (SOD1,2), and thioredoxin (TRX) for antioxidants. Examples of enzymes that detoxify are as follows: prostaglandin reductase, alcohol dehydrogenase, cytochrome P450, and carbonyl dehydrogenase [32]. On the other hand, Klotho also activates antioxidants such as CAT, peroxiredoxins (Prx-2 and 3), SOD-2, and thioredoxin reductase (Trxrd-1) [34], in addition to calcium balance regulators [35]. Vitamin D may have a key impact on calcium ion balance maintenance inside cells by regulating the expression of many mechanisms of calcium signals, such as plasma membrane Ca^2+^-ATPase (PMCA), calbindin, Na^+^/Ca^2+^ exchanger (NCX1), parvalbumin, and transient receptor potential V members 5 and 6 (TRPV5,6) [32,33,36]. In addition, vitamin D can control inflammation as it inhibits the expression of cytokines that lead to inflammation by inhibiting the translocation of nuclear factor-кB (NF-кB) since it works to induce the expression of a gene for both transcription of tumor necrosis factor-alpha (TNF-α) and interleukin (IL-1 and IL-6) [37]. Vitamin D also induces anti-inflammatory substances such as interleukin-10 (IL-10). Other research suggests that vitamin D has a more significant function in producing anti-inflammatory substances [38]. Several earlier research works have shown the relationship between vitamin D and RAAS, proving the relevance of this relationship in blood pressure regulation [39]. Another study demonstrated that vitamin D deficiency leads to negative regulation of RAAS, that is, excessive gene expression and enhanced activation of renin and angiotensin II, which elevates systolic and diastolic blood pressure, which, in turn, leads to left ventricular enlargement [28]. RAAS, which is known to have a significant role in maintaining blood pressure, has been modified in earlier research to remove both the VDR gene and the alpha-hydroxide enzyme, leading to a rise in renin gene expression and activation of the enzymes renin and angiotensin II. Thus, PTH activity increases dramatically, which is known to influence the mechanism of hypertension by raising intracellular calcium levels [40]. It is also thought that PTH might block the interchange of sodium and hydrogen, which adds to the impact of spontaneous hypertension [41]. In addition, the increase in PTH hormone produces an increase in insulin resistance and inflammation, both of which contribute to the development of atherosclerosis [42]. With regard to the proliferation and differentiation of cells, vitamin D affects the activity of several genes that are essential for the activation of numerous signaling pathways, as well as the equilibrium of the extracellular matrix, hence preserving the development and remodeling of blood vessels [43]. Vitamin D suppresses increases in the number of vascular smooth muscle cells by decreasing the induction of c-myc RNA, which further regulates negative factors of cell proliferation, such as transforming growth factor beta (TGF-β) inhibitor and plasminogen activator inhibitor-1 (PAI-1) [26,44,45]. In addition, vitamin D regulates matrix proliferation and endothelial cell homeostasis through matrix metalloproteinases (MMPs) and also vascular endothelial growth factor (VEGF) [46]. MMPs influence angiogenesis and vascular remodeling by destroying extracellular matrix proteins, while it is well known that VEGF increases endothelial cell proliferation and migration and promotes vascular growth and angiogenesis [47]. Immune and inflammatory cells play crucial roles in all kinds of CVD, including atherosclerosis, and VDR has been identified in the majority of immune cells, as well as dendritic cells, activated T cells, and macrophages [48]. In general, 1,25(OH)2D regulates inflammatory and immunological responses by maintaining their physiological limitations [49]. In Figure 3, an increase in plasma LDL concentration and accumulation due to high cholesterol as a result of metabolic disorder and lifestyle causes the first stage of the atherosclerosis process, which begins gradually in the presence of factors responsible for this occurrence. This leads to the accumulation of LDL in the subendothelial cavities of blood vessels. As the amount of reactive oxygen species in the body rises, oxidative stress occurs, causing LDL to be modified and oxidized. This is seen as a precursor to plaque inflammation and a potential predictor of CVD. In the subsequent phase, it induces the synthesis of pro-inflammatory cytokines, which initiate an immunological response and entice immune cells such as monocytes and T cells to infiltrate the affected region. Macrophages develop into foam cells because they expand after ingesting Ox-LDL particles. The contents of the foam cells are discharged into the subendothelial regions of the blood arteries once the cells have died. The long-term persistence of this process will result in the establishment of a damaged area. Ultimately, this wounded area is converted into plaques, which gradually grow, resulting in their rupture. This, in turn, disturbs the presence of smooth muscle cells and collagen in the surrounding region since the lining above it becomes more vulnerable and liable to rupture [50,51,52]. Damage to the endothelium causes the cells of the endothelium to lose the capacity to create blood clotting inhibitors. This results in an increased risk of blood clots entering the lumen of the artery. Because of this, a clot develops on the artery wall, which ultimately results in either a heart attack or a stroke [53]. Increased inflammation, which means increased synthesis of inflammatory cytokines, and diminished expression and activation of VDR all arise from a lack of vitamin D, which increases the signaling of inflammatory signaling cascades, which, in turn, leads to decreased collagen production, fibrosis, oxidative stress, inflammation, heightened infection susceptibility, and weakened defense mechanisms [54,55]. Finally, vitamin D is crucial for a number of functions, including bone metabolism, the regulation of blood pressure, and calcium and glucose balance. In addition, scientists are continuing to examine the role that vitamin D plays in maintaining a healthy body.

## 4. Importance of Vitamin D in Cardiovascular Disease

The relationship between vitamin D and CVD remains unclear and controversial. Vitamin D insufficiency is closely related to metabolic syndromes, including RAAS, raised levels of pro-inflammatory cytokines, calcium homeostasis, high lipid levels, and hyperglycemia owing to insulin resistance. All of these factors contribute to the progression of cardiovascular disease. The effect of vitamin D levels was linked to an improved risk of CVD in a previous study, which is related to heart diseases such as coronary artery disease, as the study concluded there is a clear association between vitamin D insufficiencies and decreased coronary flow, which affects endothelial function and can worsen atherosclerosis [56]. Thus, it has been established that increased vitamin D levels help coronary artery flow and improve endothelial function. Vitamin D insufficiency has been linked to the possibility of myocardial infarction [57]. In addition, previous research has shown that a rise in vitamin D levels over the recommended levels may help reduce the risk of myocardial infarction, particularly in those with little sun exposure [58]. Myocardial hypertrophy is one of the potential unfavorable outcomes of vitamin D insufficiency in heart disease. Previous research has shown a substantial correlation between low blood vitamin D levels and poor cardiac muscle performance, defined as the failure of the organ to pump enough blood through the body, due to left ventricular hypertrophy [59]. A previous study demonstrated that low vitamin D levels are associated with heart damage, which leads to acute myocardial infarction (AMI), and there is also a link between vitamin D deficiency and low triiodothyronine (LT3) syndrome in AMI. Moreover, vitamin D supplementation was used to restore the thyroid gland and normalize vitamin D deficiency. In fact, it counteracts acute imbalances in TH levels caused by oxidative stress in AMI, in addition to the effect normally associated with vitamin D [60,61]. In fact, low vitamin D levels are significant in causing weak heart muscle, even if the ejection fraction is normal. Previous studies reported that infants with dilated cardiomyopathy and subpar cardiac function may have a history of severe hypocalcaemia due to rickets [62]. When treated with vitamin D and calcium supplementation, symptoms of heart failure disappeared, and dilated cardiomyopathy and cardiac function both showed significant improvement. It has been noted that vitamin D has a significant impact on myocarditis, a leading contributor to infants developing dilated cardiomyopathy, due to the existence of a relationship between cardiomyopathy and rickets [63]. In terms of fibrosis that occurs in the heart and causes heart failure, the results of previous studies indicate the possibility that vitamin D is essential due to the presence of polymorphisms of vitamin D receptors that may affect fibrosis in patients with systolic heart failure [64]. Many clinical states, including cardiac remodeling, provide evidence of a connection between vitamin D receptor genetics and disease development [65]. Previously conducted experiments have pointed to the significance of the vitamin D signaling pathway in the progression of heart failure following exposure to a variety of conditions that have an effect on the heart [66]. Heart failure is the ultimate significant outcome of all of the diseases that have been discussed so far that are associated with heart disease and its relationship to vitamin D deficiency. The effects of vitamin D on heart disease have been discussed at length, but the discovery of VDR in the lining of blood vessels suggests that vitamin D may also have positive effects on other vascular disorders [58]. Previous research has shown that vitamin D may be a factor in a number of vascular diseases, including arterial disease, which includes aneurysms, peripheral artery disease, arterial calcification, hypertension, and atherosclerosis [67]. According to research, a lack of vitamin D is connected to the severity of arterial disorders such as aneurysms, and the mechanism that causes occlusive diseases such as aneurysms is distinct from that which causes atherosclerosis [24]. A graded inverse connection may exist between vitamin D levels and the diameter of the abdominal aortic aneurysm according to the findings of one study, which found an association between low levels of vitamin D and the severity of abdominal aortic aneurysms [68,69]. Another study also found that the severity of the aneurysms increased in proportion to the patient’s vitamin D levels [70]. In addition, inflammation plays a significant role in the formation of aneurysms, and the impact that vitamin D has is well understood. Since it is an important anti-inflammatory drug, it has the potential to lessen inflammation and its development. In addition, previous research has linked low vitamin D levels in the blood with peripheral artery disease (PAD) and chronic limb ischemia. These observations raise the potential that vitamin D deficiency may have an impact on the growth of more progressive PAD [71]. There is mounting evidence that phosphate toxicity has a significant impact on vascular calcification, and there is also growing evidence to suggest that low vitamin D levels are related to widespread vascular calcification [72]. This is a phenomenon very similar to the vascular disease that is observed in patients who have chronic kidney disease [73]. According to the findings of a previous study, an imbalance between vasoconstriction and vasodilation may be caused by a number of different factors. One of these factors may be a vitamin D deficiency, which could cause vasoconstriction, resulting in elevated hypertension [74]. Furthermore, there is a documented relationship between vitamin D insufficiency and hypertension, metabolic syndrome, and cardiovascular disease risk factors. Obesity is a major risk factor for CVD due to its relationship with the lipid profile and, particularly, increased blood levels of LDL [75]. In obese individuals, perivascular adipose tissue (PVAT) releases substances that affect atherosclerosis and the proliferation of smooth muscle cells, thereby altering systolic performance [76]. Endothelial cells are protected by vitamin D, which reduces endoplasmic reticulum stress and oxidative stress and, in turn, reduces the risk of atherosclerosis. In addition, environmental factors, age, gender, socioeconomic position, and dietary diversification all have substantial and variable influence on vitamin D levels [77]. To learn how vitamin D affects the structure and function of muscle cells in the cardiovascular system, more reliable experimental models have been used. These models were created through the selective deletion of the VDR gene from a myocardial cell as described in a previous study [78]. Using a different method, findings from other research indicate that there was an influence on heart metabolism, morphology, and function in animals defective in 1,25(OH)2D synthesis because of the selective omission of the alpha1-(OH) ase gene [79]. Moreover, in relevant experimental models, associations between vitamin D deficiency and oxidative stress, active metabolic abnormalities, cardiac inflammation, cardiomegaly, and changes in the left ear and left ventricle, as well as fibrosis, apoptosis, and systolic dysfunction, were found. There was a correlation between these findings, inflammatory indicators, and increased cytokine release. However, many of the trials and meta-analyses that have been performed on humans in an effort to demonstrate the cardiovascular benefits of vitamin D supplementation have been inconclusive, having never yielded important findings indicating major adverse cardiovascular events [80,81]. Therefore, further research is required to confirm the connection between vitamin D and CVD, in addition to the purported advantages of taking vitamin D supplements to enhance one’s cardiovascular system.

## 5. Conclusions

Multiple biological systems are affected by vitamin D, including the cardiovascular system. According to preliminary findings, vitamin D may play a role in the prevention and regulation of cardiovascular disease. Vitamin D exerts its cardiovascular effects by decreasing renin–angiotensin–aldosterone system activity, re-regulating calcium homeostasis, decreasing or increasing levels of inflammatory and anti-inflammatory agents, increasing antioxidant levels, controlling proliferation and discrimination, and acting as an anticoagulant. Strong evidence suggests that metabolic syndromes, such as stress, diabetes, oxidative stress, and obesity, may be a contributory factor owing to their high incidence and negative impact on vitamin D status and cardiovascular mortality. Future clinical studies and evidence-based therapy recommendations may benefit from a better knowledge of vitamin D’s biological mechanisms. Although prospective clinical trials to regulate the cardio-protective benefits of dietary vitamin D supplementation or VDR activators are still required, vitamin D insufficiency may act as a novel CVD severity marker and prediction. Nevertheless, the significance of vitamin D in cardiovascular disease is inconclusive; further research is required.

## Data Availability

Not applicable.
